# Caries in Horses and Dogs’ Teeth

**Published:** 1889-04

**Authors:** 


### CARIES IN HORSES AND DOGS' TEETH.

In a recent letter received from Dr. Miller, Berlin, he says that he has been studying decay in the teeth of animals, and that he has succeeded in making some beautiful preparations which show identically the same appearance found in caries of human teeth. The micro-organisms principally concerned are micrococci. Dr. Miller is a most indefatigable worker and is always reaching out into new fields. His book on Dental Pathology is nearly ready for the press. We look forward to its perusal with pleasure.

